# Feasibility and accuracy of the ASERT digital questionnaire in mood tracking for a longitudinal research study on bipolar disorder

**DOI:** 10.1016/j.xjmad.2025.100145

**Published:** 2025-08-23

**Authors:** Isaac Lynch, Gail I.S. Harmata, Ercole John Barsotti, Jess G. Fiedorowicz, Aislinn J. Williams, Cari Linkenmeyer, Sarah Smith, Spencer Smith, Jenny Gringer Richards, Jeffrey D. Long, Soňa Sikorová, Eduard Bakstein, John A. Wemmie, Vincent A. Magnotta

**Affiliations:** a Carver College of Medicine, The University of Iowa, United States; b Department of Radiology, The University of Iowa, United States; c Department of Psychiatry, The University of Iowa, United States; d Iowa Neuroscience Institute, The University of Iowa, United States; e Department of Epidemiology, The University of Iowa, United States; f Department of Psychiatry, University of Ottawa, Ontario, Canada; g School of Epidemiology and Public Health, University of Ottawa, Ontario, Canada; h Ottawa Hospital Research Institute, Ontario, Canada; i Department of Biostatistics, University of Iowa, IA, United States; j Mindpax s.r.o., Prague, Czech Republic; k National Institute of Mental Health, Klecany, Czech Republic; l Department of Cybernetics, Faculty of Electrical Engineering, Czech Technical University in Prague, Prague, Czech Republic; m Department of Molecular Physiology and Biophysics, The University of Iowa, IA, United States; n Department of Neurosurgery, The University of Iowa, IA, United States; o Veterans Affairs Medical Center, Iowa City, IA, United States; p Department of Biomedical Engineering, The University of Iowa, IA, United States

**Keywords:** Bipolar disorder, Mood tracking, Longitudinal studies, Depression, Mania

## Abstract

**Background:**

It is challenging for bipolar disorder (BD) studies to capture multiple mood states within a participant at in-person visits. Mood tracking could aid scheduling, but evaluation is usually done using clinical assessments inconvenient for participants to undergo often. However, frequent assessments are necessary to capture dynamic mood changes typical of BD. The Aktibipo Self-Rating Questionnaire (ASERT) is a simple, self-report mood survey. We examined the utility of collecting the ASERT weekly to assess mood changes and schedule follow-up visits.

**Methods:**

Sixty-one participants with BD completed the ASERT and were administered the Montgomery-Åsberg Depression Rating Scale (MADRS) and Young Mania Rating Scale (YMRS) during a baseline visit. Participants were then sent weekly text messages with an ASERT survey link. If participants exhibited at least a 5-point (later 8-point) change from baseline on either the mania or depression subscale, they were called and administered the MADRS or YMRS. A 10-point change on either phone-delivered clinical scale prompted a follow-up visit. Associations between ASERT subscales and clinical scales were evaluated using Spearman’s correlation and robust regression.

**Results:**

Mean completion rate was 94.8 % and median completion time was 67 s. The ASERT depression and mania subscales correlated with the MADRS and YMRS at baseline and all follow-up time points. Our screening method aided scheduling, with 15 of 19 participants exhibiting a 10-point change or greater on the MADRS and/or YMRS at Visit 2.

**Conclusions:**

The ASERT can be feasibly deployed to track mood and can help schedule follow-up assessments in BD longitudinal studies.

## Highlights

• Obtaining in-person data across mood states in bipolar disorder is difficult.
• We demonstrate a feasible way to track mood to schedule follow-up visits.
• We used the Aktibipo Self-Rating Questionnaire (ASERT) to track mood.
• The ASERT corresponded to clinical scales at all time points tested.
• Change in the weekly ASERT from baseline predicted depression change at follow-up.

## Graphical Abstract

**Figure:**
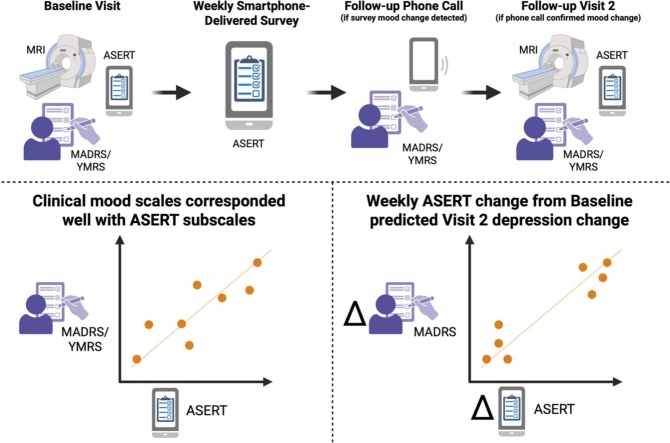
Graphical Abstract Abbreviations: MRI, magnetic resonance imaging; ASERT, Aktibipo Self-Rating Questionnaire; MADRS, Montgomery-Åsberg Depression Rating Scale; YMRS, Young Mania Rating Scale.

## 1 Introduction

Bipolar disorder (BD) is a chronic mental disorder, characterized by symptoms of depression and mania or hypomania that are often episodic [1]. An ongoing challenge in BD research is accurately tracking mood in research participants over the course of a longitudinal research study. This is especially problematic when the study design also involves assessment of individuals in different mood states during in-person visits, such as for neuroimaging or cognitive testing. If a fixed time interval is used to schedule in-person visits, it is unlikely that distinct mood states will be captured in each participant without sampling across many in-person visits. Similarly, if sampling at a fixed interval (e.g., three months), it is likely that some mood changes will occur during the intervening time between visits and those would not be captured. Continuing fixed-interval visits indefinitely until a mood change is captured in a given participant is generally not feasible or cost-effective. To better capture abnormal mood states and their effects without raising participant burden and costs associated with numerous in-person visits, it would be ideal to accurately track participant mood over time to optimize scheduling of visits to correspond with changes in mood.

Two gold standard scales for assessing mood in BD are the Montgomery-Åsberg Depression Rating Scale (MADRS) [2] and Young Mania Rating Scale (YMRS) [3]. These scales are administered by a clinician or a trained rater, typically in-person, and can take between 15 and 30 min each to administer. This makes it difficult and burdensome both for the participants and the research staff to assess mood using these scales. Therefore, there is a need in the field for an assessment which can be rapidly administered on a regular basis (e.g., at least weekly) which also has good validity with these gold standard measures of mood.

A method to address this gap is use of brief, smartphone-based, self-report mood scales. Smartphone delivery allows such scales to be readily deployed in a format convenient for participants. Previous studies have demonstrated that such questionnaires can achieve statistically significant correlation to standard clinical mood assessments [4], [5], [6], [7] and can achieve high completion rates [8], [9], [10], [11]. One example is the Aktibipo Self-Rating Questionnaire (ASERT), a 10-question survey designed for mood tracking in BD [6]. It asks respondents to consider their mood symptoms in the previous week and to rate their level of agreement on a 5-point scale. The ASERT includes 4 questions that ask about depression symptoms, 4 questions about mania symptoms, and 2 about other mood symptoms not specific to depression or mania. The prompts are shown in Supplemental Table 1. The ASERT has key advantages, such as rapid completion time, coverage of both dimensions of mood symptoms of BD (i.e., depression and mania), and amenability to delivery and completion on smartphone devices [6]. Most critically, an initial validation study using the original Czech version of the prompts demonstrated that patients’ scores on the depression- and mania-specific subscales on the ASERT correlated with patient’s MADRS and YMRS scores, respectively [6]. Given the high validity of the original ASERT when compared to the MADRS and YMRS scales and its other benefits, we chose to utilize this questionnaire as a weekly screening tool for a longitudinal neuroimaging research study evaluating functional and metabolic imaging changes associated with mood in BD.

The present analysis evaluates whether weekly administration of our modified English version of the ASERT was able to meet the following criteria: (1) achieved at least 80 % adherence from respondents in filling out weekly ASERT surveys via text-message delivery; (2) demonstrated statistically significant correlation between depression- and mania-specific ASERT subscales and their scores on MADRS and YMRS assessments, respectively; and (3) successfully screened for changes in mood for the purpose of optimizing the scheduling of follow-up visits in our ongoing neuroimaging study.

## 2 Methods

**Fig. 1:**
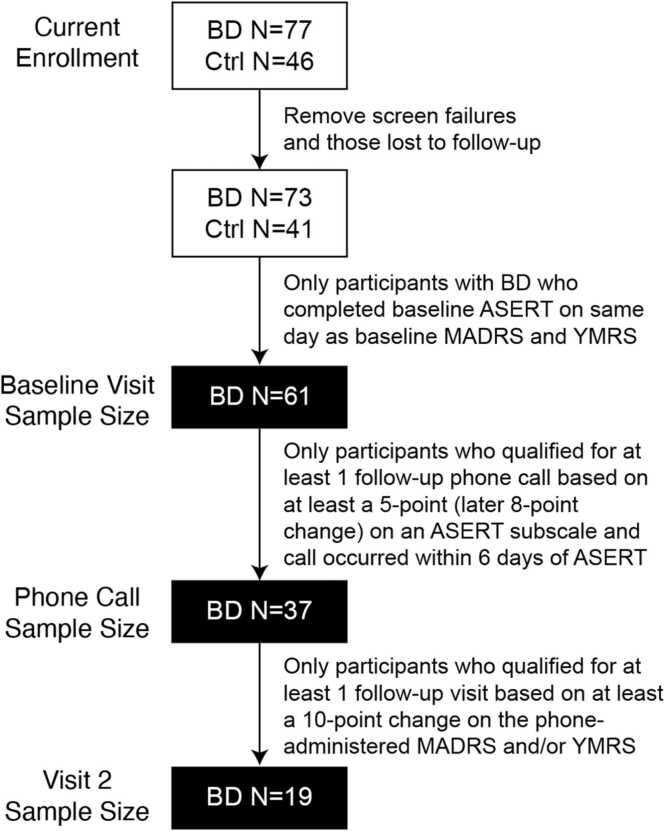
Participant diagram of current mood tracking study. Participants with bipolar disorder (BD) and control participants (Ctrl) were enrolled in an ongoing longitudinal study, but only data from participants with BD was used for this analysis, as this group was measured with weekly mood tracking using the Aktibipo Self-rating questionnaire (ASERT).

Following University of Iowa Institutional Review Board approval, individuals with and without BD (either I or II) were recruited into an ongoing longitudinal neuroimaging study that sought to assess functional and metabolic changes associated with mood. Potential participants were screened and excluded from the study if they had comorbid neurological disorders, lifetime loss of consciousness for more than 10 min, current treatment for mood disorders other than BD, pregnancy, or MR contraindications. Participants who completed this initial screening and then provided written informed consent were enrolled into the study and evaluated using the Structured Clinical Interview for DSM Disorders to verify a psychiatric diagnosis of BD (or lack thereof, for putative control participants). Only individuals with BD were followed for weekly mood tracking and eligible for mood-change follow-up visits. At the time of this analysis, 77 participants with BD had been enrolled into the study with 61 completing an initial baseline visit on the same day as completing the ASERT (Fig. 1). Notably, the study was designed to follow each participant for two years, but as the study is still ongoing, no participant had completed their end-of-study visit at the time of this analysis; thus, only 19 participants with BD had two or more available timepoints, and we had insufficient sample size to examine visits beyond Visit 2 at this time.

During the baseline visit for the study, participants completed numerous assessments including demographic information, a brief psychiatric history, medication list, cognitive and emotional assessment using the NIH Toolbox [12], [13], suicide history using the Columbia Suicide Severity Rating Scale [14], the Beck Anxiety Inventory (BAI) [15], the YMRS [3], the MADRS [2], and a variety of neuroimaging scans. During this visit, participants with BD also shared their cellphone numbers to receive a weekly text message via their smartphones to complete the Aktibipo Self-Rating Questionnaire (ASERT; [6]) scale (Table 1). The ASERT questionnaire is owned by Mindpax (Prague, Czech Republic), which encourages its use for research purposes but requires consent from the company. Notably, we slightly modified the prompts of the ASERT scale from the original English translation for clarity and to emphasize that respondents should consider symptoms over the past week (changes are highlighted Supplemental Table 1). To complete the ASERT scale, Twilio was used to send a text message containing a link which took them to a REDCap (Research Electronic Data Capture) survey [16], [17]. Each question of the ASERT scale was scored from 0 to 4 scale: 0 =Strongly Disagree;1 = Disagree; 2 = Neither agree nor disagree; 3 =Agree; and 4 =Strongly Agree. The responses were used to calculate depression and mania subscales scores calculated from 4 questions each (with possible sums of 0–16), as well to calculate versions of depression and mania subscales scores with the two non-specific items added to each (with possible sums of 0–24). Importantly, participants with BD were not required to be euthymic at baseline; whatever mood scale ratings they reported at baseline were considered their initial reference state for the purpose of the study.

**Table 1:** Demographics for participants with a qualifying baseline visit (N = 61). Participants had to complete the ASERT survey on the same day as their in-person baseline neuroimaging visit to be included.

Variable	Metric	BD (N = 61)
Age (years)	Mean (SD)	39.3 (14.0)
	Range	18.3–70.5
Sex	Female:Male (% Female)	44:17 (72 %)
Subjective SES	Mean (SD)	4.9 (1.9)
	N-Miss	2
	Range	1–9
Self-reported Race	American Indian or Alaska Native	1 (2 %)
	Asian	1 (2 %)
	Black/African American	3 (5 %)
	White	51 (85 %)
	Multiracial	2 (3 %)
	Unknown	2 (3 %)
	N-Miss	1
Self-reported Ethnicity	Hispanic or Latino	2 (3 %)
	Not Hispanic or Latino	54 (89 %)
	Unknown	5 (8 %)
Bipolar Disorder Subtype	BD I:BD II (% BD I)	47:14 (77 %)
History of Suicide Attempt	True:False (% True)	30:31 (49 %)
History of Hospitalization	True:False (% True)	43:18 (70 %)
Estimated Symptom Burden:		
% Time Depressed	Mean (SD)	37.7 (23.3)
	N-Miss	2
	Range	0.0–95.0
% Time Manic	Mean (SD)	19.1 (14.6)
	N-Miss	2
	Range	0.0–60.0
% Time Euthymic	Mean (SD)	43.2 (26.3)
	N-Miss	2
	Range	0.0–98.0

Abbreviations: ASERT = Aktibipo Self-rating questionnaire; BD = bipolar disorder; N-Miss = number missing; SD = standard deviation; SES = socioeconomic status

Once participants with BD enrolled into the two-year longitudinal study, they received a new link each Tuesday to the weekly ASERT scale. If participants had a change of 5 or more points on either the mania or depression sub-scales of the ASERT from their last in-person visit (later increased to 8 or more points, based on study experience and participant feedback to reduce burden), study staff phoned the participants to administer either the YMRS or MADRS scales based on the sub-scale that had changed within the same week period. Thus, the subsequent YMRS or MADRS scales were administered within six days of the ASERT scale. Notably, when administering these phone assessments, MADRS question 1 (apparent sadness) and YMRS question 10 (appearance) were excluded since the scales were administered over the phone. Therefore, the YMRS and MADRS scores reported from phone calls are called “modified” in the subsequent analysis. If participants had a modified MADRS score and/or modified YMRS score change by 10 more points from their baseline MADRS and/or YMRS, they were invited back to participate in a mood change follow-up visit that included functional and metabolic imaging. At this follow-up visit, the ASERT, YMRS, and MADRS were again completed on the same day to confirm that mood had not changed from the follow-up phone call and to establish new baseline scores. The resetting of the baseline was done to avoid bringing in participants more than once during the same mood episode; however, for this paper, we only considered the initial baseline visit (Visit 1) and first mood scale change (Visit 2) to simplify the analysis. To incentivize continued participation in the study, participants were compensated $5 USD each time they filled out a survey if they had completed at least 2 surveys in a month.

Participant data was stored in a secure REDCap database. R/RStudio was used for analysis [18], [19]. Participant survey adherence rates were determined by dividing the number of surveys they had completed by the number of weeks they were enrolled in the study. As the ASERT wording was slightly modified for clarity, we checked that each subscale remained internally consistent by calculating Cronbach’s alpha using the R *psych* package [20]. Due to a lack of normality and presence of potential outliers, particularly among YMRS scores, Spearman correlation and robust regression were used to evaluate the relationships between ASERT subscale scores and clinical assessment scores. Spearman correlation is a nonparametric ranked correlation test and does not assume normality [21]. Similarly, robust linear regression is resilient against non-normality and outliers thus resulting in more accurate beta coefficients [22]. These analyses were performed at three time points: baseline (Visit 1; N = 61), first follow-up phone call (N = 37), and first follow-up visit (Visit 2; N = 19). Finally, we also used robust regression to test whether the change from baseline in the weekly ASERT that elicited a phone call successfully predicted change in clinical mood scales from baseline when participants were brought in for Visit 2. Change in clinical mood scales was defined as at least a 10-point change from baseline on either the MADRS or YMRS at Visit 2. We used a delta to define a mood change rather than a set threshold for each clinical mood scale because (a) there is not a universally agreed-upon cutoff for “euthymia”, “depression”, or “mania” using these scales, and (b) we wished to consider mood symptoms using more nuanced assessments rather than discrete categories of “euthymic”, “depressed”, and “manic”. Spearman correlations were performed with base R, while robust regression with robust F-test was performed using the *MASS* and *sfsmisc* packages [23], [24]. Other R packages used for data cleaning, graphing, and demographic statistics included those from the *tidyverse* [25], *fuzzyjoin* [26], *ggprism* [27], and *arsenal* [28].

## 3 Results

### 3.1 Cohort demographics

At the time of analysis, 61 participants with BD had completed a baseline neuroimaging visit with clinical mood scales on the same day as the ASERT survey (see study flowchart in Fig. 1). Demographics for this sample can be seen in Table 1. In brief, the mean age was 39.3 years (SD = 14.0 years, range: 18.3–70.5 years). The sample was predominately female (72.1 %) and White (85.0 %). Around half of the sample had a prior suicide attempt (49.2 %), and most of the sample had been previously hospitalized (70.5 %). Self-estimated symptom burden over the last 10 years (or since diagnosis) varied widely between participants; however, participants tended to endorse more time spent in depression (mean = 37.7 %, SD = 23.3 %) and euthymia (mean = 43.2 %, SD = 26.3 %) than in mania (mean = 19.1 %, SD = 14.6 %).

Of these 61 participants, 37 qualified for at least one follow-up phone call due to changes in mood in the ASERT survey. Nineteen of those participants were brought back in for at least one follow-up visit because the mood changes suggested by the ASERT were verified to be large enough during their follow-up phone calls, as measured with either the modified MADRS or modified YMRS (see methods). Demographics for these sub-samples are shown in Supplemental Tables 2 and 3. Both sub-samples showed largely similar demographics to the overall sample, although the sample at Visit 2 was slightly older and had a slightly higher ratio of females to males than the original sample.

### 3.2 The ASERT was completed regularly and quickly

**Fig. 2:**
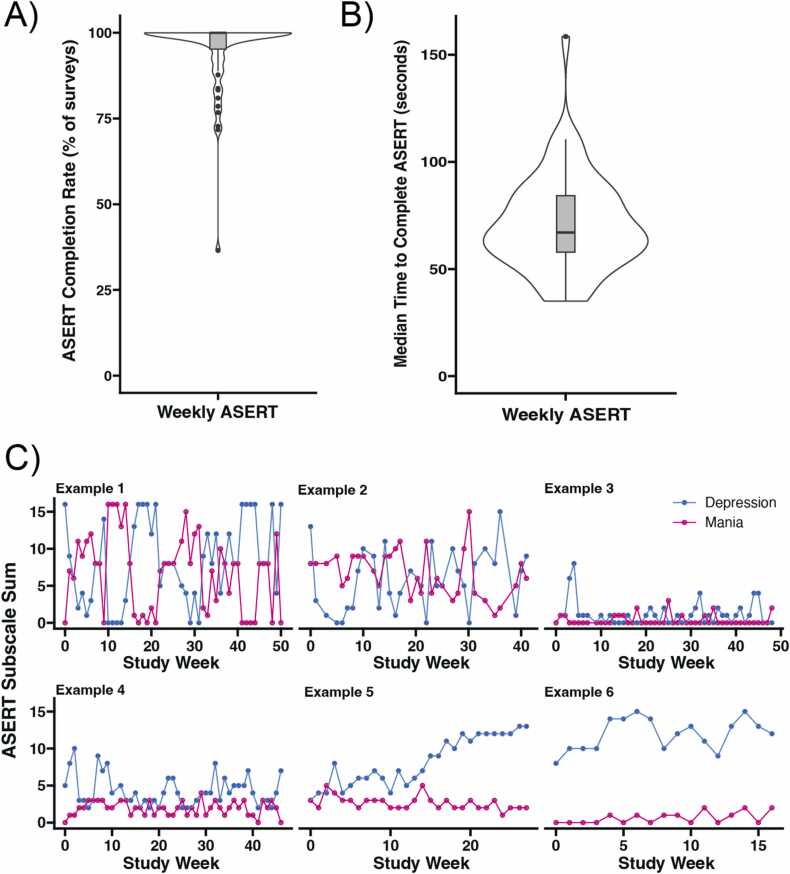
The Aktibipo Self-rating questionnaire (ASERT) survey was completed regularly and quickly and appeared to capture changes in mood over time in participants with bipolar disorder enrolled in the study’s text-message-delivered mood tracking for at least 10 weeks (N = 56). (A) We achieved a mean completion rate of 94.8 % across these participants, with most participants having a completion rate of 100 %. (B) The ASERT was generally completed very quickly, with a median completion time of 67 s. (C) Examples of ASERT mood tracking across six different participants, with the ASERT depression subscale in blue and the ASERT mania subscale in magenta.

Of the 61 participants, 56 had been enrolled in the study long enough to receive 10 or more opportunities to respond to a weekly ASERT and had a smartphone for text-based delivery of the survey links. The mean completion rate for these 56 individuals was 94.8 % (Fig. 2A) with 60.7 % of participants having responded to all surveys. Median time to complete the weekly ASERT survey was 67 s (Fig. 2B). Examples of dynamics in the ASERT survey mood ratings are shown in Fig. 2C.

### 3.3 The ASERT subscales were internally consistent and corresponded to MADRS and YMRS scores at the baseline visit

**Fig. 3:**
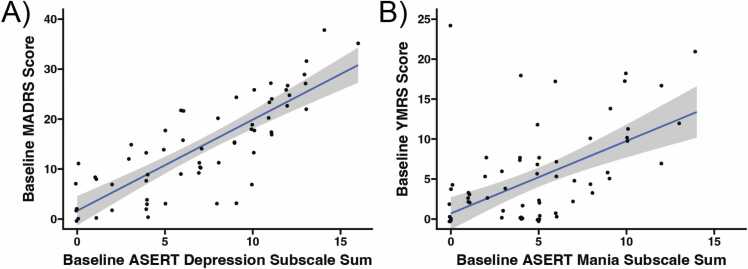
The Aktibipo Self-rating questionnaire (ASERT) subscales corresponded well with clinical mood scales at the study baseline visit in individuals with bipolar disorder (N = 61). (A) Baseline ASERT depression subscale scores were highly correlated with baseline Montgomery-Åsberg Depression Rating Scale (MADRS) scores (Spearman’s rho = 0.812, p < 0.001). (B) Baseline ASERT mania subscale scores were correlated with the baseline Young Mania Rating Scale (YMRS) scores (Spearman’s rho = 0.496, p < 0.001).

The ASERT prompts used had revised wording as shown in Supplemental Table 1; despite these minor changes, subscale consistency remained high, as Cronbach’s alphas for the ASERT depression and mania subscales at the baseline visit were 0.88 and 0.80, respectively. MADRS scores and ASERT depression subscale scores were significantly correlated at the baseline visit (Spearman’s rank correlation rho = 0.812, p < 0.001; Fig. 3A). Robust regression indicated a 1.8-point increase in MADRS score for each 1-point increase in the ASERT depression subscale (Fig. 3A). Subjects’ total YMRS scores and ASERT mania subscale scores had a lower but still significant correlation at study baseline (Spearman’s rank correlation rho = 0.496, p < 0.001) (Fig. 3B). Robust regression indicated a 0.9-point increase in YMRS score for each 1-point increase in the ASERT mania subscale (Fig. 3B). Similar results were seen if non-specific ASERT questions were added to each ASERT subscale (Supplemental Figure 1). Correlation and robust regression results from all analyses in the manuscript can be found in Supplemental Table 4.

### 3.4 The ASERT subscales corresponded to modified MADRS and YMRS scores at first follow-up phone call

**Fig. 4:**
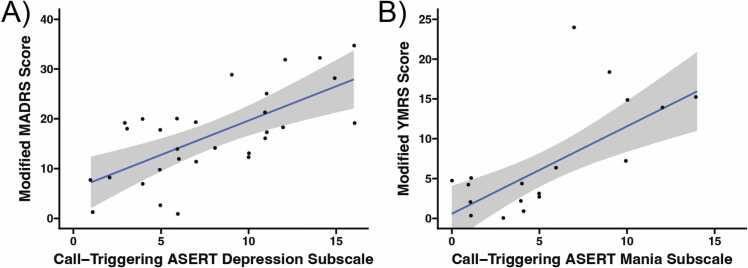
The Aktibipo Self-rating questionnaire (ASERT) subscales reported during weekly mood tracking corresponded well with modified clinical mood scales during the first follow-up phone call for each participant (N = 37). Each participant was administered a phone-friendly version of the Montgomery-Åsberg Depression Rating Scale (MADRS) and/or Young Mania Rating Scale (YMRS), depending on which ASERT subscales showed a sufficient delta from their last visit (baseline). (A) The weekly ASERT depression subscale scores were highly correlated with the modified MADRS scores during the first follow-up phone call (with the item “Apparent Sadness” being skipped; Spearman’s rho = 0.624, p < 0.001). (B) The weekly ASERT mania subscale scores were highly correlated with the modified YMRS scores during the first follow-up phone call (with the item “Appearance” being skipped; Spearman’s rho = 0.672, p = 0.002).

Thirty-seven participants qualified for at least one follow-up phone call based on a change in one or both of their ASERT subscales from baseline (either a change of at least 5 or at least 8, see Methods). We termed the ASERT survey instance that prompted a phone call a “call-triggering ASERT”. At the first follow-up phone call for each participant, the modified MADRS (excluding the “Apparent Sadness” item) and/or the modified YMRS (excluding the “Appearance” item) was administered, depending on which ASERT subscale(s) had changed. The call-triggering ASERT depression subscale was significantly correlated with the subsequent phone call’s modified MADRS score (Spearman’s rank correlation rho = 0.624, p < 0.001; Fig. 4A). Similarly, the call-triggering ASERT mania subscales were significantly correlated with the subsequent phone call’s modified YMRS score (Spearman’s rank correlation rho = 0.672, p = 0.002; Fig. 4B).

### 3.5 The ASERT subscales corresponded to MADRS and YMRS scores at Visit 2

Nineteen participants were brought back for a follow-up visit (Visit 2) to capture a mood change for our neuroimaging study. At this time point, MADRS scores and ASERT depression subscale scores were again significantly correlated (Spearman’s rank correlation rho = 0.888, p < 0.001; Supplemental Figure 2A). Subjects’ total YMRS scores and ASERT mania subscale scores were also correlated at Visit 2 (Spearman’s rank correlation rho = 0.536, p = 0.018) (Supplemental Figure 2B). Using robust regression, similar beta coefficients were found in both analyses as with the baseline visit, with a 1.9-point increase in MADRS score with each 1-point increase in the ASERT depression subscale and a 1.1-point increase in YMRS score with each 1-point increase in the ASERT mania subscale.

### 3.6 The change in weekly ASERT successfully predicted a change in mood at Visit 2

**Fig. 5:**
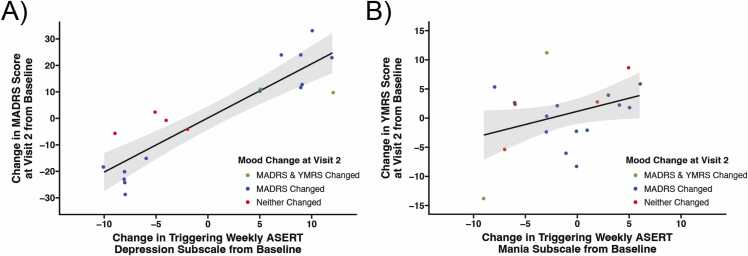
Weekly mood tracking with Aktibipo Self-rating questionnaire (ASERT) surveys facilitated capture of mood change from baseline during Visit 2 (N = 19). Fifteen out of 19 participants with a Visit 2 had change in Montgomery-Åsberg Depression Rating Scale (MADRS) and/or Young Mania Rating Scale (YMRS) score of at least 10 points (78.9 %). Most participants had a change in the MADRS only (N = 13), while two participants changed on both MADRS and YMRS from their baseline scores. No individuals had only a change in YMRS only. (A) The change in the ASERT depression subscale from baseline during weekly mood tracking predicted the subsequent change in MADRS score from baseline during Visit 2 using robust regression (p < 0.001). (B) The change in the ASERT mania subscale from baseline during weekly mood tracking did not significantly predict change in YMRS score during Visit 2 using robust regression (p = 0.164).

For the purposes of this manuscript, only the first follow-up visit for a qualifying participant (Visit 2) was analyzed. Out of the 19 participants brought in for a Visit 2, 15 participants (78.9 %) had a change in MADRS and/or YMRS scores of at least 10 points. Notably, most mood changes were in the MADRS scale only (N = 13), while two participants had changes in both MADRS and YMRS scales. This was consistent with our finding that the change from baseline detected in the call-triggering ASERT depression subscale significantly predicted the MADRS score change from baseline at Visit 2 using robust regression (p < 0.001; Fig. 5A). Meanwhile, the call-triggering ASERT mania subscale did not significantly predict the YMRS score change at Visit 2 using robust regression (p = 0.164, Fig. 5B).

## 4 Discussion

While obtaining frequent and accurate mood information in BD research studies may be challenging, our study indicates that use of the ASERT survey resulted in very high levels of participant adherence and that mood measurements correlated strongly with contemporaneous gold standard ratings of mood. Furthermore, using change in the ASERT to guide the scheduling of follow-up visits for the neuroimaging portion of our study was largely successful, with almost 80 % of screened participants meeting our definition of a mood change. These findings suggest that the ASERT survey is a promising method to track mood over longitudinal studies in patients with BD with minimal participant burden.

In our study, of the 56 individuals with at least 10 opportunities to complete the weekly digital ASERT, our mean adherence rate was 94.8 %. This adherence rate is higher than many BD studies involving mood-tracking [5], [6], [29], [30], [31] but in line with others [8], [9], [10]. Two possible contributors to our high adherence rate are the brevity of the 10-question ASERT and the compensation ($5 USD) we provided for survey completion. The ASERT link was texted directly to participants’ phones and took on average less than 70 s to complete, minimizing participant burden. Other studies have reported over 90 % average adherence using smartphone-delivered assessments [8], [9], [10] with delivery frequency varying from multiple times a day to weekly. The assessments in these studies were always concise and easy to complete, suggesting that mood assessment brevity increases adherence. However, the original ASERT survey validation study only observed 78.1 % adherence over a time interval of 18 months [6] when compensation was not provided for survey completion. We suspect that our participant compensation maintained the high level of adherence in the present study. A study on survey length and compensation suggests that maximum participation is achieved with using short surveys coupled with compensation [32], suggesting our high adherence rate is in line with what should be expected given our study design. Nevertheless, as our study is still ongoing, it is possible our currently strong survey completion rates will diminish over the course of the study, especially as individuals have more opportunities to experience mood episodes that could disrupt survey adherence. Full study completion will help us to fully evaluate the success of our survey administration method.

In addition to high adherence, we also observed that the ASERT subscales scores aligned well with the standard scales used to assess mood in BD. At each timepoint evaluated (baseline, follow-up phone call, and Visit 2), the ASERT depression and mania subscales correlated well with the MADRS and YMRS scales, respectively. The MADRS tended to have higher correlation coefficients with the ASERT depression subscale than the YMRS with the ASERT mania subscale (e.g., at baseline, the Spearman rho’s were around 0.8 vs. 0.5, respectively). These observations are consistent with the findings of the original ASERT validation study using the Czech version of the scale [6]. The lower correlation coefficient associated with mania-related scales could be due to several factors. Firstly, it is well-established that mania is more difficult to track from self-report, possibly due to reduced insight during a manic episode [33]. Secondly, while the YMRS covers psychotic symptoms associated with mania, the ASERT mania questions do not. Thus, we observed several instances in which participants who endorsed “hearing voices” or other positive psychotic symptoms had a high YMRS score but did not have a correspondingly high ASERT mania subscale score. Therefore, it is unsurprising that the correlation for YMRS vs. the ASERT mania subscale would be weaker than the comparison for depression.

While our findings were in line with the general observations of the ASERT validation study, there were some differences observed as well. For example, our correlation coefficients appeared higher than what was previously reported; for example, for MADRS vs. ASERT depression subscale, Anýž et al. reported a weighted Pearson coefficient of 0.5 [6], while we reported Spearman rho of around 0.8. Additionally, our beta coefficients differed from those reported in the original validation study; for example, we observed around a 1.4-point increase in MADRS with each 1-point increase in the ASERT depression subscale with nonspecific questions added during the baseline visit (see Supplemental Figure 1A**,** Supplemental Table 4), while the original study observed around a 0.9-point increase in MADRS for each 1-point increase in the ASERT depression scale with nonspecific questions added [6]. While it is possible that these differences are real, these apparent discrepancies may be misleading, as different statistical methods were used in each case (weighted Pearson correlation vs Spearman correlation and linear mixed effects modeling vs robust regression). Furthermore, we also analyzed our timepoints separately to avoid weighting for uneven repeated measures, which resulted in very different sample sizes. Thus, direct comparison of the coefficients is inappropriate in this case. If differences do exist, possible differences could include language used (Czech vs. American English), prompt wordings (present vs. past week), different study populations (Czech vs. midwestern U.S.), and different raters for the MADRS and YMRS scales.

In terms of using the ASERT for scheduling follow-up visits, our method of using change on the weekly ASERT to schedule mood-change visits appeared to work well. The method consisted of comparing the weekly ASERT subscale scores to a participant’s baseline score, calling them if one or more subscales changed and delivering a modified MADRS/YMRS over the phone, and bringing in those who appeared to have a change of at least 10 points on either clinical scale. Fifteen of the 19 participants brought in for a Visit 2 had a change of at least 10 points on the MADRS and/or YMRS on the day of the visit. The remaining four participants were enrolled early in the study and brought back under the original ASERT subscale change criteria of 5 points, but three would not have met the later, more stringent 8-point cutoff; thus, it is unsurprising that these three showed sub-threshold changes on both the MADRS and YMRS. Furthermore, two of the four participants without a mood change at Visit 2 displayed frequent mood fluctuations, which may have led to mood changes not being captured in time due to scheduling delays. While the final screening method was generally successful, it is possible that the ASERT screening method for research could be refined further for even better change detection depending on the type of mood symptoms experiencing change. While we do not intend to further modify our ASERT change cutoffs further at this point in the study, future studies using the ASERT for screening mood may wish to test differing cutoffs or other criteria by subscale. It is also important to note that the screening method used in this study was not intended for use in clinical tracking, mood episode prediction, or intervention, but rather to capture a variety of mood states in each person during our neuroimaging research study. While the ASERT itself may be useful clinically [6], the present work does not evaluate our screening method for clinical applications, and additional studies would be needed to explore such use cases.

Notably, our screening method using the ASERT detected far more changes in depression than in mania when bringing in participants for Visit 2. Out of 19 participants, we detected 13 with changes in depression only, and 2 with changes in both depression and mania; no participants had changes in mania only. There are several possible reasons for this. Firstly, individuals with BD tend to spend more time in depressive episodes than manic episodes [34], which may have reduced the likelihood of capturing a change in manic symptoms over the study course. Secondly, since our sample had a smaller range of change from baseline in YMRS scores as compared to MADRS scores at Visit 2, this likely limited our ability to detect any statistical relationship with change on the ASERT mania subscale. Thirdly, mania is associated with reduced insight into one’s own symptoms [33], which could make self-report accuracy on the ASERT less reliable and make changes harder to detect. Fourthly, while the ASERT mania subscale did correlate with the YMRS in our study, it wasn’t as strong of a correlation as the ASERT depression subscale with the MADRS, in part because the ASERT manic subscale doesn’t cover psychotic symptoms as described above. Finally, if a participant is in the midst of a manic episode, this could affect survey adherence, ability to be contacted, and ability to be scanned; for example, we had an instance in which a participant missed an ASERT survey due to being hospitalized, which the participant explained at a later date. Therefore, it remains unclear whether our screening method using the ASERT is truly less effective at detecting changes in mania versus depression. As our study continues to enroll and follow participants, we will gain greater insight into this possibility.

The current study has several limitations. Firstly, our definition of a mood change was based on what we considered a relatively large change on either the MADRS or YMRS (10 points), but this exact threshold was somewhat arbitrary. Future studies are needed to determine if a different change threshold is more clinically relevant, and whether this change threshold varies depending on the clinical scale used. Secondly, as the use of a mood changed based on a delta value (rather than a discrete cutoff) is not commonly used, it is unclear how this method compares to using discrete mood state cutoffs to define a mood state change. While we intend to explore stratifying our imaging data by symptom severity once our study has completed, future studies should directly test different definitions of mood change against each other to see if there is any impact on number of follow-up visits or neuroimaging outcomes. Thirdly, the Visit 2 sample size is limited. This is partly because the study is still ongoing and enrolling participants, but also because we are limiting follow-up visits to putative mood changes, which depends entirely on the mood stability of our enrolled participants. However, as we will be following each participant for two years and obtaining baseline and end-of-study visits for every participant, our longitudinal coverage will greatly expand. Indeed, by the end of the study, we will have information about mood fluctuations on a weekly basis for up to 150 participants with BD. Nevertheless, for the current analysis, it is still a relatively small sample. The small sample size also prevented us from evaluating potential differences between BD I and BD II in this manuscript, which we hope to revisit once our sample size has grown. Fourthly, our results may not be representative of the population at large, as the sample thus far is from a single site and is predominantly White and female. As we continue to increase our sample size, we will explore potential differences in the ASERT screening method between demographic groups. Finally, while our multi-stage method of scheduling mood change follow-up visits appeared to work well in reducing participant burden and study costs, this came at a cost of less available data. For example, as we only call participants for phone screening if they meet change criteria on at least one ASERT subscale, we do not have weekly MADRS and YMRS data for all participants. This prevents us from examining correlation of the ASERT subscale changes week-by-week to clinical scale data. Thus, project priorities should be considered carefully prior to implementing this scheduling method for research.

In conclusion, the present analyses suggest that the ASERT is a simple, low-burden survey that can be used to screen for mood changes in longitudinal studies of BD and may be especially effective for detecting changes in depressive symptoms. The ASERT may thus be a valuable tool for researchers to track mood in BD over time and to schedule follow-up visits for the most efficient use of resources.

## Funding

This work was supported by 10.13039/100000002National Institutes of Health [NIMH grants R01MH111578 and R01MH125838, 10.13039/100006108NCATS grant UL1TR002537, 10.13039/100000002NIH Instrumentation grants S10OD025025 and S10RR028821, and NIH CTSA grant UM1TR004403]. John A. Wemmie was also supported by the 10.13039/100000738U.S. Department of Veterans Affairs [Merit Award], and Gail I. S. Harmata was supported by the National Institutes of Health [NIMH grant T32MH019113]. Eduard Bakstein was supported by the Ministry of Health of the Czech Republic, grant nr. NU23–04–00534 and the ERDF-Project Brain dynamics, No. CZ.02.01.01/00/22_008/0004643. None of the funding sources influenced any aspect of study design, data collection, analysis, or writing of the manuscript.

## Declaration of Competing Interest

The ASERT questionnaire is associated with Mindpax (Mindpax s.r.o., Prague, Czech Republic) and their proprietary tools. Our study was given permission to use the ASERT for research purposes but was not funded by Mindpax. Two coauthors on this study are affiliated with Mindpax (Soňa Sikorová and Eduard Bakstein). Jeffrey D. Long recently received direct financial compensation from the following entities for board membership work (unrelated to the present manuscript): Alexion, Alnylam, NIH (DSMB), Harness, PTC, Roche, and uniQure. Jeffrey D. Long also recently received direct financial compensation from the following entities for consultancy (unrelated to the present manuscript): Jazz, Latus Bio, LifeEDIT, LoQus23, Prilenia, Rgenta, Sage, Skyhawk, Spark, Vertex, and Wave.

## Footnotes

Appendix A Supplementary data associated with this article can be found in the online version at doi:10.1016/j.xjmad.2025.100145.

## References

[1] Chakrabarti S. (2022). Bipolar disorder in the international classification of Diseases-Eleventh version: a review of the changes, their basis, and usefulness. World J Psychiatry.

[2] Montgomery S.A., Asberg M. (1979). A new depression scale designed to be sensitive to change. Br J Psychiatry.

[3] Young R.C., Biggs J.T., Ziegler V.E., Meyer D.A. (1978). A rating scale for mania: reliability, validity and sensitivity. Br J Psychiatry.

[4] Sagorac Gruichich T., David Gomez J.C., Zayas-Cabán G., McInnis M.G., Cochran A.L. (2021). A digital self-report survey of mood for bipolar disorder. Bipolar Disord.

[5] Tsanas A., Saunders K.E., Bilderbeck A.C., Palmius N., Osipov M., Clifford G.D. (2016). Daily longitudinal self-monitoring of mood variability in bipolar disorder and borderline personality disorder. J Affect Disord.

[6] Anýž J., Bakštein E., Dally A., Kolenič M., Hlinka J., Hartmannová T. (2021). Validity of the aktibipo Self-rating questionnaire for the digital self-assessment of mood and relapse detection in patients with bipolar disorder: instrument validation study. JMIR Ment Health.

[7] Faurholt-Jepsen M., Vinberg M., Frost M., Christensen E.M., Bardram J.E., Kessing L.V. (2015). Smartphone data as an electronic biomarker of illness activity in bipolar disorder. Bipolar Disord.

[8] Ryan K.A., Babu P., Easter R., Saunders E., Lee A.J., Klasnja P. (2020). A smartphone app to monitor mood symptoms in bipolar disorder: development and usability study. JMIR Ment Health.

[9] Faurholt-Jepsen M., Ritz C., Frost M., Mikkelsen R.L., Margrethe Christensen E., Bardram J. (2015). Mood instability in bipolar disorder type I versus type II-continuous daily electronic self-monitoring of illness activity using smartphones. J Affect Disord.

[10] McKnight R.F., Bilderbeck A.C., Miklowitz D.J., Hinds C., Goodwin G.M., Geddes J.R. (2017). Longitudinal mood monitoring in bipolar disorder: course of illness as revealed through a short messaging service. J Affect Disord.

[11] Pellegrini A.M., Huang E.J., Staples P.C., Hart K.L., Lorme J.M., Brown H.E. (2022). Estimating longitudinal depressive symptoms from smartphone data in a transdiagnostic cohort. Brain Behav.

[12] Salsman J.M., Butt Z., Pilkonis P.A., Cyranowski J.M., Zill N., Hendrie H.C. (2013). Emotion assessment using the NIH toolbox. Neurology.

[13] Weintraub S., Dikmen S.S., Heaton R.K., Tulsky D.S., Zelazo P.D., Bauer P.J. (2013). Cognition assessment using the NIH toolbox. Neurology.

[14] Posner K., Brown G.K., Stanley B., Brent D.A., Yershova K.V., Oquendo M.A. (2011). The Columbia–Suicide severity rating scale: initial validity and internal consistency findings from three multisite studies with adolescents and adults. Am J Psychiatry.

[15] Beck A.T., Epstein N., Brown G., Steer R. (1993). Beck anxiety inventory. J Consult Clin Psychol.

[16] Harris P.A., Taylor R., Thielke R., Payne J., Gonzalez N., Conde J.G. (2009). Research electronic data capture (REDCap)—A metadata-driven methodology and workflow process for providing translational research informatics support. J Biomed Inform.

[17] Harris P.A., Taylor R., Minor B.L., Elliott V., Fernandez M., O'Neal L. (2019). The REDCap consortium: building an international community of software platform partners. J Biomed Inform.

[18] Team Posit (2023). Posit Software.

[19] Core Team R. (2023). Version 4.3.1 [software].

[20] Revelle W. (2024). R package version 2.4.12 [software].

[21] Spearman C. (1904). The proof and measurement of association between two things. Am J Psychol.

[22] Wager T.D., Keller M.C., Lacey S.C., Jonides J. (2005). Increased sensitivity in neuroimaging analyses using robust regression. NeuroImage.

[23] Venables W.N., Ripley B.D. (2002).

[24] Maechler M. (2024). Utilities from 'Seminar fuer Statistik' ETH Zurich. R package version .1- [software].

[25] Wickham H., Averick M., Bryan J., Chang W., McGowan L., François R. (2019). Welcome to the tidyverse. J Open Source Softw.

[26] Robinson D. fuzzyjoin: Join Tables Together on Inexact Matching. R package version 0.1.6 [software]. 2020.

[27] Dawson C. (2024). Ggprism: a 'ggplot2' extension inspired by 'GraphPad prism. R Package Version 1 0 5.

[28] Heinzen E., Sinnwell J., Atkinson E., Gunderson T., Dougherty G. arsenal: An Arsenal of 'R' Functions for Large-Scale Statistical Summaries. R package version 3.6.3 [software]. 2021.

[29] Bilderbeck A.C., Atkinson L.Z., McMahon H.C., Voysey M., Simon J., Price J. (2016). Psychoeducation and online mood tracking for patients with bipolar disorder: a randomised controlled trial. J Affect Disord.

[30] Bopp J.M., Miklowitz D.J., Goodwin G.M., Stevens W., Rendell J.M., Geddes J.R. (2010). The longitudinal course of bipolar disorder as revealed through weekly text messaging: a feasibility study. Bipolar Disord.

[31] Schwartz S., Schultz S., Reider A., Saunders E.F.H. (2016). Daily mood monitoring of symptoms using smartphones in bipolar disorder: a pilot study assessing the feasibility of ecological momentary assessment. J Affect Disord.

[32] Kost R.G., de, Rosa J.C. (2018). Impact of survey length and compensation on validity, reliability, and sample characteristics for Ultrashort-, Short-, and Long-Research participant perception surveys. J Clin Transl Sci.

[33] Cassidy F. (2010). Insight in bipolar disorder: relationship to episode subtypes and symptom dimensions. Neuropsychiatr Dis Treat.

[34] Tondo L., Vázquez G.H., Baldessarini R.J. (2017). Depression and mania in bipolar disorder. Curr Neuropharmacol.

