# Supplementary material for: Feasibility and accuracy of the ASERT digital questionnaire in mood tracking for a longitudinal research study on bipolar disorder
Source: J Mood Anxiety Disord. 2025 Aug 23;12:100145. doi: 10.1016/j.xjmad.2025.100145 (PMC12445695; doi:10.1016/j.xjmad.2025.100145)
Supplement: Supplementary file 1 — Supplementary material [file mmc1.docx]

**Supplemental Table 1.** Prompts for the ASERT were modified slightly from their translation from the original version published by Anýž et al. (2021) for greater clarity and to emphasize reporting over the past week. Participants responded to each item on a 5-point scale, from Strongly Disagree (0) to Strongly Agree (4); this was also a revised wording from the original scale (which ranged from “I do not agree” to “I completely agree”).

| ASERT Subscale | Item No. | Original English Prompt^1^ | Revised Prompt |
| --- | --- | --- | --- |
| Depression | 1 | I feel sad, downhearted | In the past week, I felt sad, downhearted. |
|  | 2 | I do not enjoy anything, and nothing pleases me | In the past week, I did not enjoy anything, and nothing pleased me. |
|  | 3 | I have no energy | In the past week, I had no energy. |
|  | 4 | I feel gloomy and pessimistic about the future | In the past week, I felt gloomy and pessimistic about the future. |
| Mania | 5 | I feel unusually great, optimistic | In the past week, I felt unusually great, optimistic. |
|  | 6 | I have excess energy | In the past week, I had excess energy. |
|  | 7 | My thinking is very fast, others cannot keep up with me | In the past week, my thinking was very fast, others could not keep up with me. |
|  | 8 | I need to sleep less than usual | In the past week, I needed to sleep less than usual. |
| Nonspecific | 9 | I feel restless, tense | In the past week, I felt restless, tense. |
|  | 10 | I cannot focus | In the past week, I could not focus. |

**^1^**From Anýž et al., 2021 (PMID 34383689).

Abbreviations: ASERT = Aktibipo Self-rating questionnaire

**Supplemental Table 2.** Demographics for participants with at least one follow-up phone call triggered by a change on the weekly ASERT mood subscales (N = 37). Participants had to receive a follow-up phone call within 6 days of an ASERT mood change to be included.

| Variable | Metric | BD (N=37) |
| --- | --- | --- |
| Age (years) | Mean (SD) | 41.5 (13.8) |
|  | Range | 23.9 - 70.5 |
| Sex | Female:Male (% Female) | 29:8 (78%) |
| Subjective SES | Mean (SD) | 4.4 (2.0) |
|  | N-Miss | 1 |
|  | Range | 1 - 9 |
| Self-reported Race | American Indian or Alaska Native | 1 (3%) |
|  | Asian | 0 (0%) |
|  | Black/African American | 1 (3%) |
|  | White | 32 (89%) |
|  | Multiracial | 1 (3%) |
|  | Unknown | 1 (3%) |
|  | N-Miss | 1 |
| Self-reported Ethnicity | Hispanic or Latino | 1 (3%) |
|  | Not Hispanic or Latino | 33 (89%) |
|  | Unknown | 3 (8%) |
| Bipolar Disorder Subtype | BD I:BD II (% BD I) | 29:8 (78%) |
| History of Suicide Attempt | True:False (% True) | 21:16 (57%) |
| History of Hospitalization | True:False (% True) | 28:9 (76%) |
| Estimated Symptom Burden: |  |  |
| % Time Depressed | Mean (SD) | 36.7 (19.7) |
|  | N-Miss | 1 |
|  | Range | 1.0 - 70.0 |
| % Time Manic | Mean (SD) | 19.1 (13.7) |
|  | N-Miss | 1 |
|  | Range | 0.0 - 50.0 |
| % Time Euthymic | Mean (SD) | 44.1 (23.7) |
|  | N-Miss | 1 |
|  | Range | 10.0 - 98.0 |

Abbreviations: ASERT = Aktibipo Self-rating questionnaire; BD = bipolar disorder; SD = standard deviation; N-Miss = number missing; SES = socioeconomic status

**Supplemental Table 3.** Demographics for participants with at least one follow-up visit prompted by a mood change verified during a follow-up phone call (N = 19). During the follow-up phone call, modified forms of the MADRS (without the “Apparent Sadness” item) and the YMRS (with the “Appearance” item) were administered, and a change of 10 points or more points on these scales was considered a mood change.

| Variable | Metric | BD (N=19) |
| --- | --- | --- |
| Age (years) | Mean (SD) | 43.7 (13.0) |
|  | Range | 25.4 - 69.3 |
| Sex | Female:Male (% Female) | 16:3 (84%) |
| Subjective SES | Mean (SD) | 3.9 (1.8) |
|  | Range | 1.0 - 7.0 |
| Self-reported Race | American Indian or Alaska Native | 1 (5%) |
|  | Asian | 0 (0%) |
|  | Black/African American | 0 (0%) |
|  | White | 17 (90%) |
|  | Multiracial | 0 (0%) |
|  | Unknown | 1 (5%) |
| Self-reported Ethnicity | Hispanic or Latino | 1 (5%) |
|  | Not Hispanic or Latino | 16 (84%) |
|  | Unknown | 2 (11%) |
| Bipolar Disorder Subtype | BD I:BD II (% BD I) | 15:4 (79%) |
| History of Suicide Attempt | True:False (% True) | 12:7 (63%) |
| History of Hospitalization | True:False (% True) | 14:5 (74%) |
| Estimated Symptom Burden: |  |  |
| % Time Depressed | Mean (SD) | 36.0 (18.5) |
|  | N-Miss | 1 |
|  | Range | 2.5 - 70.0 |
| % Time Manic | Mean (SD) | 24.8 (14.3) |
|  | N-Miss | 1 |
|  | Range | 2.5 - 50.0 |
| % Time Euthymic | Mean (SD) | 39.2 (22.4) |
|  | N-Miss | 1 |
|  | Range | 10.0 - 95.0 |

Abbreviations: ASERT = Aktibipo Self-rating questionnaire; BD = bipolar disorder; SD = standard deviation; N-Miss = number missing; SES = socioeconomic status

**
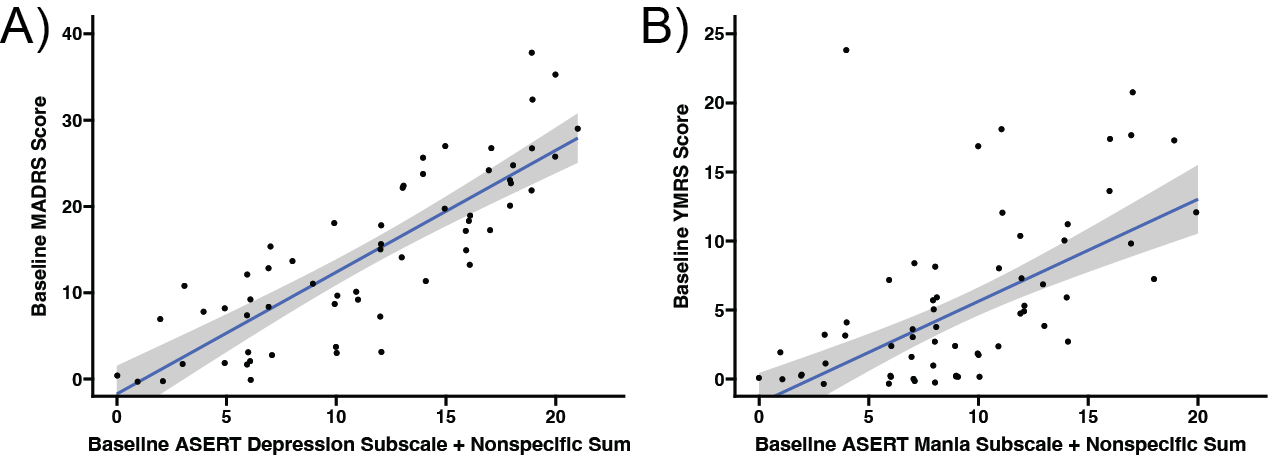
**

**Supplemental Figure 1.** The Aktibipo Self-rating questionnaire (ASERT) subscales sums with nonspecific questions added corresponded well with clinical mood scales at the study baseline visit in individuals with bipolar disorder (N = 61). (A) Baseline ASERT depression subscales with nonspecific questions added were highly correlated with baseline MADRS scores (Spearman’s rho = 0.862, p < 0.001). (B) Baseline ASERT mania subscales with nonspecific questions added were correlated with the baseline YMRS scores (Spearman’s rho = 0.626, p < 0.001).


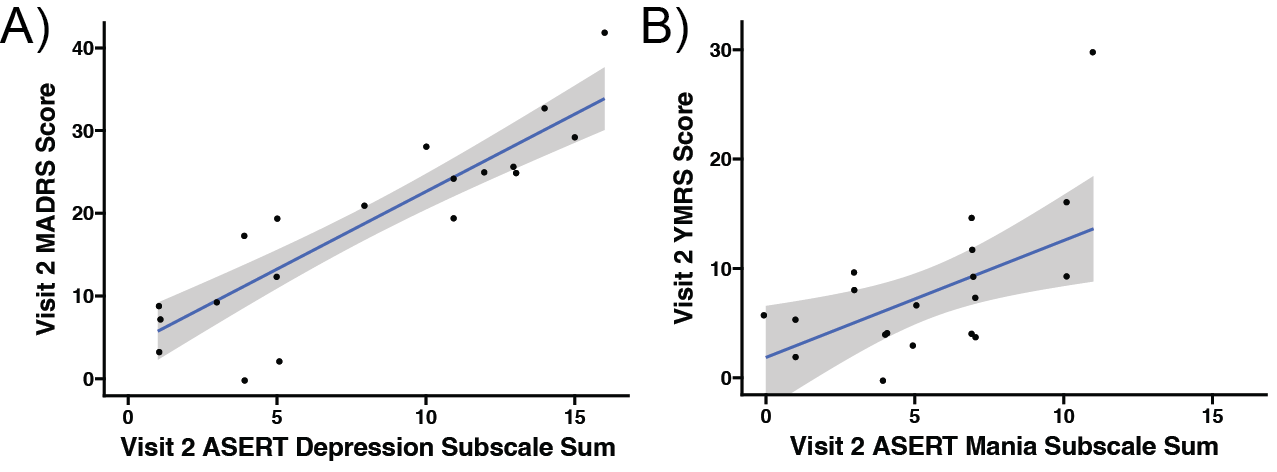


**Supplemental Figure 2.** The Aktibipo Self-rating questionnaire (ASERT) subscales corresponded well with clinical mood scales at the first mood change follow-up visit (Visit 2) in individuals with bipolar disorder (N = 19). (A) ASERT depression subscales were highly correlated with MADRS scores at Visit 2 (Spearman’s rho = 0.888, p < 0.001). (B) ASERT mania subscales were correlated with the YMRS scores at Visit 2 (Spearman’s rho = 0.536, p = 0.018).

**Supplemental Table 4.** Spearman correlation and robust regression results for paper figures 3-5 and supplemental figures 1 and 2.

| Figure | Description | Statistical Test | Test Statistic | p-value | Spearman's rho or  Beta coefficient (SE) |
| --- | --- | --- | --- | --- | --- |
| Fig. 3A | Baseline: ASERT depression subscale vs. MADRS | Spearman Corr. | S = 7097 | p < 0.001 | Spearman's rho: 0.812 |
|  |  | Robust Regression | F = 113.18 | p < 0.001 | Beta: 1.822 (0.172) |
| Fig. 3B | Baseline: ASERT mania subscale vs. YMRS | Spearman Corr. | S = 19063 | p < 0.001 | Spearman's rho: 0.496 |
|  |  | Robust Regression | F = 34.70 | p < 0.001 | Beta: 0.906 (0.153) |
| Fig. 4A | Phone Call: call-triggering ASERT depression subscale vs. modified MADRS | Spearman Corr. | S = 1688.4 | p < 0.001 | Spearman's rho: 0.624 |
|  |  | Robust Regression | F = 22.164 | p < 0.001 | Beta: 1.378 (0.294) |
| Fig. 4B | Phone Call: call-triggering ASERT mania subscale vs. modified YMRS | Spearman Corr. | S = 318.10 | p = 0.002 | Spearman's rho = 0.672 |
|  |  | Robust Regression | F = 21.51 | p < 0.001 | Beta: 1.096 (0.240) |
| Fig. 5A | Delta from baseline: call-triggering ASERT depression vs. Visit 2 MADRS | Spearman Corr. | S = 161.25 | p < 0.001 | Spearman's rho = 0.859 |
|  |  | Robust Regression | F = 66.72 | p < 0.001 | Beta: 2.045 (0.250) |
| Fig. 5B | Delta from baseline: call-triggering ASERT mania vs. Visit 2 YMRS | Spearman Corr. | S = 771.95 | p = 0.178 | Spearman's rho = 0.323 |
|  |  | Robust Regression | F = 2.12 | p = 0.164 | Beta: 0.451 (0.306) |
| Supp. Fig. 1A | Baseline: ASERT depression plus nonspecific questions vs. MADRS | Spearman Corr. | S = 5224.6 | p < 0.001 | Spearman's rho = 0.862 |
|  |  | Robust Regression | F = 118.86 | p < 0.001 | Beta: 1.412 (0.130) |
| Supp. Fig. 1B | Baseline: ASERT mania plus nonspecific questions vs. YMRS | Spearman Corr. | S = 14141 | p < 0.001 | Spearman's rho = 0.626 |
|  |  | Robust Regression | F = 46.55 | p < 0.001 | Beta: 0.739 (0.108) |
| Supp. Fig. 2A | Visit 2: ASERT depression subscale vs. MADRS | Spearman Corr. | S = 127.78 | p < 0.001 | Spearman's rho = 0.888 |
|  |  | Robust Regression | F = 65.39 | p < 0.001 | Beta: 1.874 (0.232) |
| Supp. Fig. 2B | Visit 2: ASERT mania subscale vs. YMRS | Spearman Corr. | S = 529.21 | p = 0.018 | Spearman's rho = 0.536 |
|  |  | Robust Regression | F = 9.45 | p = 0.007 | Beta: 1.069 (0.334) |
